# The critical need for robust decision support in the era of precision cancer therapeutics

**DOI:** 10.37349/etat.2026.1002356

**Published:** 2026-01-22

**Authors:** Maurie Markman

**Affiliations:** Chinese Academy of Medical Sciences and Peking Union Medical College, China; Medical Oncology and Therapeutics Research, City of Hope Comprehensive Cancer Center, Duarte, CA 91010, USA

It is difficult to overstate the magnitude of the impact of revolutionary changes over the past several decades in our understanding of the molecular biology of cancer and the potential therapeutic implications associated with these findings. Further, the simply stunning clinical opportunities resulting from advances in technology have permitted relatively rapid evaluation of the molecular profiles of a specific patient’s tumor (both tissue and blood) at a continuing decreasing cost, making such “testing” increasingly available to a larger proportion of health systems and individuals with cancer.

However, the proliferation of validated relevant molecular targets, either in specific tumor sites (e.g., EGFR mutations in lung cancer) or agnostic to the site of origin (e.g., mismatch repair deficient advanced or metastatic cancers) and the multiple drugs developed and subsequently available to employ in the real-world clinical setting must surely challenge the large majority of oncologists in their attempts to select optimal therapy for individual patients under their care.

Consider, for example, the past and continuing evolution in the management of chronic myelocytic leukemia (CML), perhaps the poster child for the molecular revolution in cancer medicine [1]. While fundamental understanding of the biological basis for the malignancy had long been understood, it was the development of a “targeted” therapeutic (imatinib) and the subsequent landmark clinical trials that documented its effectiveness in this setting that subsequently heightened interest (along with other clinically meaningful experiences [2]) in the broader potential of precision cancer medicine [3, 4].

Further, there is perhaps no greater testament to the impact of precision medicine than reports revealing favorable “real-world” population-based outcomes resulting from the introduction of a new therapeutic into routine standard-of-care cancer management, as revealed in a study from Sweden [5].

Unfortunately, and of absolutely no surprise, the simplicity of employing this single agent (imatinib) in the treatment of CML would not continue long into the future, as there is an appropriate desire to discover ever more effective and less toxic drugs, and specifically agents that can overcome resistance either at diagnosis or with the subsequent emergence of resistance under the pressure of treatment. As a result, there are currently six tyrosine kinase inhibitors approved by the U.S. Food and Drug Administration (FDA), and specifically five which are stated to be appropriate for first-line treatment after diagnosis and five after disease progression [1].

How is a busy oncologist, and particularly a clinician who is not an expert in the management of hematologic malignancies, to decide which agents are best to employ in individual patients with CML? This question includes specific concerns with co-morbidities (e.g., mild to more severe cardiac, renal, hepatic, pulmonary dysfunction; diabetes, obesity, etc.), common in an elderly population most likely to develop this malignancy.

Also, to be considered are issues with polypharmacy, increasingly recognized as occurring in a population with potentially multiple conditions that might benefit from specific medications. How do the individual drugs interact such that this occurrence may potentially influence therapeutic efficacy or toxicity?

In the opinion of this commentator, the impact of the acceleration in the increasing number of clinically relevant targets in oncology, as revealed by the observed failure of a substantial percentage of patients to receive therapy of documented benefit, should be recognized as a matter of urgent concern.

A “real-world” example will help emphasize this point. In a recent report evaluating a proprietary deidentified electronic medical record database, investigators examined the delivery of PARP inhibitors to patients with metastatic prostate cancer and a BRCA mutation, a strategy that had been approved for administration by the FDA due to a demonstrated favorable impact on survival [6]. In this analysis, data collection was initiated three months after regulatory approval (August 2020) and continued through May 2024. Among 443 metastatic prostate patients with known BRCA 1/2 alterations, approximately one-half had *not received* a PARP inhibitor in their care during the stated time interval.

While it is not possible to make a definitive statement as to the number of individuals who would optimally have been expected to be treated with these drugs, considering issues of cost and deterioration in performance status preventing treatment, recognizing the existing therapeutic options, the observed percentage must be considered disturbingly low.

When one adds to the concern noted above for *information overload* the increasing trend for regulatory approval of “tumor-agnostic” indications based solely on molecular findings that permit therapeutic targeting independent of the site of origin [7], the challenge of expansion of our historical concepts of “pathogenic germline abnormalities” beyond current guidelines [8], the complexity associated with interpretation of “genetic variants of uncertain clinical significance” [9], and finally the almost certain eventual introduction of pharmacogenomics into standard workflow [10], it is clear the development of simple, low-cost, easy to implement and employ *decision-support solutions* must become a priority for the oncology community.

While it is important to acknowledge the efforts of several organizations in the creation of decision support tools, such as the American Society of Clinical Oncology and the National Comprehensive Cancer Network, these strategies are frequently more of a listing of options and provide limited assistance in dealing with individual “real-world” patients with specific co-morbidities and past medical histories.

Finally, one can quite realistically envision AI (artificial intelligence) in some form as a relevant component of a successful approach to this serious issue. Imagine the potential for AI in a private and secure environment to interrogate the electronic medical record of individual patients to discover relevant findings that might influence the selection of available therapeutic options. And based on the current trajectory of this impressive technology, this proposal is surely nearing objective reality.

## Abbreviations

AI: artificial intelligence
CML: chronic myelocytic leukemia
FDA: Food and Drug Administration

## Declarations

### Copyright

© The Author(s) 2026.
